# Factors Contributing to Resilience Among First Generation Migrants, Refugees and Asylum Seekers: A Systematic Review

**DOI:** 10.3389/ijph.2023.1606406

**Published:** 2023-12-11

**Authors:** Jutta Lindert, Florence Samkange-Zeeb, Marija Jakubauskiene, Paul A. Bain, Richard Mollica

**Affiliations:** ^1^ Department of Health and Social Work, University of Applied Sciences Emden/Leer, Emden, Germany; ^2^ Women’s Research Center, Brandeis University, Waltham, MA, United States; ^3^ Leibniz Institute for Prevention Research and Epidemiology–BIPS, Bremen, Germany; ^4^ Medical School, Vilnius University, Vilnius, Lithuania; ^5^ Countway Library of Medicine, Harvard Medical School, Boston, MA, United States; ^6^ Harvard Medical School, Boston, MA, United States

**Keywords:** resilience, hope, opportunities, transnational migrants, refugees

## Abstract

**Objectives:** We aimed at 1) collating and evaluating the current evidence on factors contributing to resilience of adult transnational migrants, 2) identifying methodological factors which contribute to the findings, 3) identifying and analyzing promotive and preventive factors contributing to the findings.

**Methods:** A systematic search for relevant studies published until 2021 was conducted in PubMed, PsycINFO, PTSDPubs, and Web of Science. Both, quantitative and qualitative peer-reviewed observational studies reporting on resilience and wellbeing, sense of coherence, or post-traumatic growth outcomes among transnational migrants (aged 18+). Risk of Bias was assessed using the Critical Appraisal Skills Program for qualitative studies and the Appraisal Tool for cross-sectional studies. Due to the heterogeneity of studies we did a narrative review.

**Results:** Database search yielded 3,756 unique records, of those *n* = 80 records, representing *n* = 76 studies met the inclusion criteria. The studies provided knowledge on resilience for *n* = 9,845 transnational migrants across 23 countries. All studies except two were cross sectional. N = 45 reported on resilience, *n* = 4 on Sense of Coherence and *n* = 15 on Post-Traumatic Growth. The study methods were not related to the findings. Future orientation, hope and religion/spirituality, caring for others and having opportunities were shown to be more pertinent to resilience outcomes than institutional care structures.

**Conclusion:** Our findings highlight that mental health professionals and policymakers should try to support positive perspectives for the future and encourage policies tailored towards giving refugees opportunities to work, learn and care and to help others.

## Introduction

Mental health conditions of transnational migrants (migrants and refugees) is a serious and growing public health problem. Migrant, is defined as a person who moves away from his or her place of usual residence, whether within a country or across an international border, temporarily or permanently. A refugee is defined as a person who: “owing to well-founded fear of being persecuted for reasons of race, religion, nationality, membership of a particular social group or political opinion, is outside the country of his nationality and is unable or, owing to such fear, is unwilling to avail himself of the protection of that country; or who, not having a nationality and being outside the country of his former habitual residence, is unable or, owing to such fear, is unwilling to return to it. In Africa, Article I (2) of the 1969 OAU Convention extends the refugee definition to: “every person who, owing to external aggression, occupation, foreign domination or events seriously disturbing public order in either part or the whole of his country of origin or nationality, is compelled to leave his place of habitual residence in order to seek refuge in another place outside his country of origin or nationality.” In Latin America, Conclusion III of the 1984 Cartagena Declaration, extends the refugee definition to: “persons who have fled their country because their lives, safety or freedom have been threatened by generalized violence, foreign aggression, internal conflicts, massive violation of human rights or other circumstances which have seriously disturbed public order.” [1, 2]. We use the term transnational migrants in this paper as an umbrella term including migrants, asylum seekers and refugees. According to the World Migration Report 2022 [3], in 2020, 3.6% of the world population, almost 281 million people, including 26.4 million refugees, lived outside their country of birth. Transnational migrants are often exposed during their journey to a range of risk factors for anxiety, depression, and posttraumatic stress (PTSD) (e.g., human rights violations, poverty, violence, travelling long distances under dangerous circumstances, discrimination in the host country). [4, 5] Pre-migration risk factors might include adversities such as violence and human rights violations, lack of basic needs such as food, water, housing and medical care, and separation from or loss of loved ones. Migration journey factors include exploitation and abuse [6]. Finally, resettlement factors include loss of important social roles, lack of employment, difficult living circumstances [7, 8] and discrimination. Yet, many transnational migrants display resilience [4, 9].

The negative effects of pre-migration, migration journey and resettlement adversities on mental health are well documented. Fazel et al. (2005) [10] conducted a systematic review of refugees resettled in high-income countries, and reported a prevalence of 9% for posttraumatic stress disorder (PTSD), 5% for depressive disorder, and 4% for generalized anxiety disorder, based on studies reporting on at least 200 participants. Another review covering studies published between 1987 and 2009, comprising 81,866 refugees and conflict-affected populations, reported an unadjusted weighted prevalence of 30% for PTSD and 30% for depression [4]. A recent systematic review of mental health conditions among refugees observed substantial heterogeneity of mental health outcomes [4, 11] (Kaade submitted). Some of the heterogeneity across the studies may be attributable to methodological differences (among others, measures used to assess outcomes), the changing nature of migrating populations (among others, different types of experiences), and the different coping strategies reported in the studies. While this literature contributes to knowledge on risk factors, less is known about factors that contribute to resilience and related conditions including posttraumatic growth and sense of coherence.

Without underestimating the exposure to a wide range of risk factors for mental health (e.g., poverty, violence, travelling long distances under dangerous circumstances, discrimination in the host country) [4, 5], transnational first-generation migrants show considerable levels of resilience, post-traumatic growth (PTG), and sense of coherence (SOC) [12–15] Resilience is a construct that has been conceptualized in diverse ways: as a trait, as an outcome and as a process, and is most commonly defined as “positive adaptation despite significant adversity” [9]. Accordingly, resilience refers to the dynamic process of a person successfully adapting to, or recovering from adversity [16, 17]. The resilience process may differ depending on the cultural, developmental, and historical context of individuals, and may vary across age and gender. Hence, it is a constantly changing interaction and adaption between an individual and his or her environment. Related to resilience are the concepts of Posttraumatic Growth (PTG) Sense of Coherence (SOC) and wellbeing. PTG as proposed by Tedeschi and Calhoun (1996) [18], is defined as a positive change in an individual’s life as a consequence of exposure to adversity. PTG goes beyond the absence of symptoms or return to a baseline function following adversity, and includes an adversity-induced increase in psychological benefits, such as a greater appreciation of life, improved interpersonal relationships, and re-evaluation of priorities in life [19–21]. PTG has been observed in survivors of different types of adversities [21], including the Holocaust [22], natural disasters, war and armed conflict [23]. Sense of coherence (SOC) is defined as a general orientation to life that represents the extent to which individuals (a) perceive events as structured, predictable and explicable, (b) feel able to deal with events, and (c) are willing and motivated to deal with these events [12]. According to Antonovsky, these three components are called comprehensibility, manageability, and meaningfulness, and together determine whether an individual has a strong or weak SOC. A further outcome is mental wellbeing [5], which includes a variety of facets such as self-acceptance, the establishment of close ties to other individuals, a sense of autonomy in thought and action, the ability to navigate complex environments and the pursuit of meaningful goals, and a sense of purpose in life and growth and development as a person [24].

Research on factors related to resilience, PTG, SOC and wellbeing has so far focused on factors deemed positive in Western population groups, such as openness to new experiences, optimism, extraversion [25] and social support [26, 27]. A systematic review investigating enablers of psychological wellbeing among refugees and asylum seekers (N = 16 articles with N = 1,352 participants) identified eight enablers of subjective wellbeing: social support; faith, religion and spirituality; cognitive strategies; education and training opportunities; employment and economic activities; behavioral strategies; political advocacy; and environmental conditions [28]. No systematic review so far investigated promotive and preventive factors contributing to resilience in transnational migrants, Going beyond the previous reviews, we include observational quantitative and qualitative studies investigating individual, family, and community factors associated with resilience and resilience related outcomes (PTG, SOC, and mental wellbeing). By providing a comprehensive synthesis of the current knowledge on promotive and preventive factors related to resilience in transnational migrants, our review provides empirical support for intervention programs and policy initiatives aimed at supporting and promoting resilience of transnational migrants. We expect this study to provide a suggestive direction for researchers, policymakers, and practitioners on developing strategies to promote and support resilience among transnational migrants and reduce mental health conditions. By drawing the evidence for multiple disciplines (e.g., public health, epidemiology, anthropology, sociology, medicine, psychology), it also draws attention to the importance of interdisciplinary collaboration including public health, anthropology, medicine and sociology.

## Methods

We conducted a systematic literature review of factors contributing to transnational migrants’ resilience. There is no universally agreed definition of the term “first-generation migrant.” In this review we include all those which are included in the United Nation’s definition of migrant as “an individual who is residing in a foreign country, irrespective of the causes, voluntary, or involuntary, and the means, regular or irregular; used to migrate” [29]. Additionally, we do not use any restriction on length of residence but include transnational migrants, independent of length of stay in the new country. Our systematic review is reported in accordance with the PRISMA guidelines [30]. Ethical approval was not required for this review as the data are publicly available.

### Search Strategy and Information Sources

We identified studies that examined resilience in migrants and refugees by searching the electronic databases PubMed (NCBI), PsycINFO (EBSCO), PTSDPubs (ProQuest), and the Web of Science Core Collection. The search, developed by a subject expert (JL) and an experienced medical librarian (PAB), included terms for refugees and migrants, together with a range of terms intended to capture studies under a broad definition of resilience. Under the broad definition of resilience were included studies investigating resilience, PTG, SOC or mental wellbeing. Controlled vocabulary terms were included when available, and no date or language restrictions were applied (Supplementary Appendix S1). The search was last updated on 15 July 2021. The most recent update in 2021 might be a limitation of this paper, however, it provides evidence on this population group of transnational migrants during the time period up to July 2021. The reference lists of included articles were examined for further studies of interest.

### Inclusion and Exclusion Criteria

Studies were eligible for inclusion in this review if they fulfilled the following criteria: (a) were peer-reviewed observational empirical studies, (b) involved first generation transnational migrants (including refugees and asylum seekers) aged 18 years and above, who lived in any country outside their home country, and (c) included one or more of the following as the main outcome: resilience, PTG, SOC or mental wellbeing. Studies were excluded if (a) they were intervention studies, (b) were conducted among (or included) children or adolescents younger than 18 years of age, (c) included seasonal or other specific groups of workers, (d) specifically focused on traumatized persons, and (e) investigated exclusively mental health or psychopathological (e.g., PTSD) and physical health outcomes. Studies focusing on coping strategies as a main outcome or resilience as a personal trait were also excluded. Further, studies using a single-case design (e.g., clinical case study) as well as books, book chapters, abstracts without full texts, conference proceedings, reviews, editorials, opinion statements, letters to the editor, reports, dissertations, theses and similar publications were excluded. We additionally excluded studies published before 1994 when the Diagnostic and Statistical Manual of Mental Disorders (DSM) was revised and updated.

### Study Screening and Selection

JL, MJ and FSZ screened the titles and abstracts as well as the full texts independently using Covidence [31], an online software program that enables the creation and management of systematic reviews. Each record or article was screened by at least two individuals. Any conflicts were discussed by the three reviewers until consensus was reached. The publications selected after the full text screening were then subjected to a final in-depth review. To ensure the eligibility of publications, the research team discussed each article in detail before making the decision to include it in the data extraction stage.

### Data Extraction and Coding

Data extraction was piloted on a small number of studies by the researchers independently. Data were extracted and managed in Excel spreadsheets. JL, MJ and FSZ extracted the following data from all included studies independently: authors, study design, participants (age, gender, type of population), outcome, outcome measure, confounders, confounder measures and results. The three authors then compared and discussed their outputs and thereby compiled the final data extraction tables. Where an included study was published in multiple articles, we used all outcome information. The unit of allocation remained the study, rather than the number of publications.

### Risk of Bias

MJ, FSZ and Jl assessed the risk of bias of the studies, with each study being assessed by at least two researchers. Discrepancies were discussed and resolved in consensus. Due to the variation in study designs included in this review, two separate appraisal tools were used to assess risk of bias: the Critical Appraisal Skills Program (CASP) [32] for qualitative studies, and the Appraisal Tool for Cross-sectional Studies (AXIS) [33] for quantitative studies. The CASP checklist includes 10 aspects to be recorded with a “yes,” “cannot tell,” or “no.” The total number of “yes” responses indicates the risk of bias level of the study, which can range from 0–10. We rated as high risk of bias studies scoring 0–2 yes responses, moderate risk of bias studies scoring 2–5 yes and low risk of bias those studies with more than 5 yes responses. The AXIS consists of 20 components to be recorded with “yes,” “no,” “don’t know/comment.” We stratified the studies according to potential risk of bias. We rated as low risk of bias studies scoring 0–2 yes responses, moderate risk of bias studies scoring 2–5 yes and high risk of bias those studies with more than 5 yes responses.

### Synthesis of Results

Due to the diversity across studies in relation to outcomes, settings, samples, methods, and measures, both qualitative and quantitative data were synthetized narratively. A thematic synthesis method was used. The authors coded the extracted text and identified descriptive themes. The latter were then collapsed into analytical themes through discussion within the research team.

## Results

### Characteristics of the Included Studies

**FIGURE 1:**
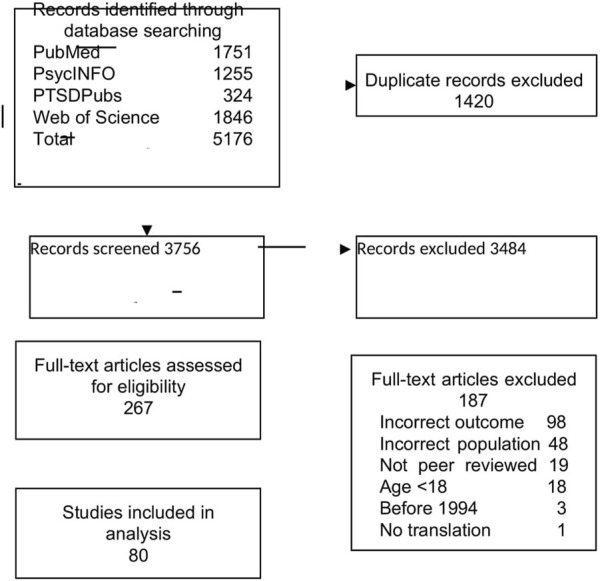
Selection of studies. Global, 2021.

A total of 3,756 unique records were identified through database searching, and 267 full-text articles were assessed for inclusion (Figure 1). Finally, we identified 80 articles, representing 77 studies, that met the inclusion criteria and were included in our analysis, providing data for n = 10,047 transnational adult migrants. Six of the studies included in the review were published before 2010, *n* = 30 studies used quantitative methods, N = 3 used mixed methods, and *n* = 44 were qualitative (Table 1). Out of the 30 quantitative studies, all except two were cross-sectional. Regarding outcomes, *n* = 45 studies reported on resilience, *n* = 4 on sense of coherence and n = 15 on post-traumatic growth and *n* = 20 on mental wellbeing, (Tables 2, 3).

**TABLE 1:** Overview of included studies (*n* = 39 qualitative, *n* = 30 quantitative, *n* = 3 mixed methods, *n* = 1 action research) Global, 2021.

Study characteristics	Qualitative (total 44)	Quantitative (total 30)
Region		
Europe	7	9
North America/Canada	11	10
Latin America/Caribbean	-	1
Africa	4	1
Australia	8	-
Asia	7	9
Population		
Refugees	34	22
Migrants	6	8
Refugees and migrants	2	1
Sample size		
<50	41	-
51–99	3	2
100–499	-	26
>500	-	2

**TABLE 2:** Studies included in the review on resilience factors in transnational migrants (migrants, refugees) Global, 2021.

Author, year, country	Country of origin	Study type	Sample: size (age range, mean / years, range), gender (%, *n*); status	Sampling procedure	Years, mean / in host country
Abraham et al., 2018, Norway [34]	Eritrea	Qual.	N = 18 (range: 18-60 years), female; refugees	Purposive	1-8
Ai et al., 2007, USA, [35]	Kosovo	Quant.	N = 50 (mean: 33, SD = 12, 17-69), 46% female (*n* = 23), 54% male (*n* = 27); refugees	Convenience	-
Aikawa and Kleyman, 2019, USA [36]	Southeast Asia, Africa, Asia	Quant.	N = 90 (mean: 31.22), 56% female (*n* = 50), 46% male (*n* = 40); refugees	Purposive	Mean: 5.12 (SD = 7.58)
Akinsulure-Smith, 2017, USA [37]	Cameroon, CAR, Gabon, Guinea, Liberia, Mali, Senegal, Sierra Leone	Qual.	N = 38 (mean: 43 years, SD = 16, 19-68), 47% female (*n* = 18), 53% male (*n* = 20); refugees and migrants	Purposive	1-37
Alduraidi et al., 2020, Jordan [38]	Syria	Quant.	N = 151 (mean: 31.3, SD = 10), 73.5% (*n* = 111 female), 26.5% (*n* = 40) males; refugees	Convenience	-
Areba et al., 2018, USA [39]	Somalia	Quant.	N = 156 (mean: 21, SD = 2.3, range 18-30), 75% (*n* = 117) females, 25% (*n* = 39) males, refugees	Convenience	-
Atari-Khan, 2021, USA [40]	Syria	Qual.	N = 8 (mean: 37, 27-59), five female, three male; refugees	Purposive	11 months – 3, 3 years
Baird, 2012; Baird and Boyle, 2012, USA [41, 42]	Sudan	Qual.	N = 10 (mean: 34.4; 25-44), female; refugees	Purposive	2-11 (m = 6.6)
Babatunde-Sowole et al., 2020, Australia [43]	West - Africa	Qual.	N = 21, 18+, female; migrants	Convenience, snowball	>12 months
Braun-Lewensohn et al., 2019, Greece [44]	Syria	Quant.	N = 111 (mean: 41.01, SD = 11.42, 19-70) female; refugees	Convenience	1 month+
Cengiz et al., 2019, Turkey [45]	Syria	Quant.	N = 310: *n* = 38.1% (*n* = 118) (18-29), 32.9% (*n* = 102) (30-39), 29% (*n* = 90) 40+; 47.1% (*n* = 146) female, 52.9% (*n* = 164) male; refugees	Convenience	3+ years, (50.6%), 1-2 years (49.4%)
Cetrez et al., 2021, Sweden [46]	Iraq	Quant.	N = 410 (18+), 46.8% (*n* = 192, mean: 34.27, SD = 14.27) female, 53.2% (*n* = 218, mean: 39.98, SD = 16.14) male; refugees	Convenience	Moved 2000-2013
Christopher, 2000), USA [47]	Ireland	Quant.	N = 100 (18+, mean: 32, SD = 5.2), 73% (*n* = 73) female, 27% (*n* = 27) male; migrants	Purposive	Emigration after 1980
Civan Kahve et al., 2020, Turkey [48]	Iraq	Quant.	101 (18+), 51.5 (*n* = 52) female, 48.5% (*n* = 49) male; refugees	Convenience	Mean 16.3 ± 11.1 months
Copping et al., 2010, Australia [49]	Sudan	Qual.	N = 15 (mean: 32.67, SD = 8.54, range 19-49), 47% (*n* = 7) female, 53% (*n* = 8) male; refugees	Purposive, snow-ball	3 months – five years
Corley and Sabri, 2021, USA [50]	Eritrea, Sudan, Uganda, Ethiopia, Kenya, Congo, Ghana	Qual.	N = 39 (mean: 39.9, SD = 9.5); refugees /migrants	Purposive, snow-ball	Mean 11.1 (SD = 8.21)
Demir, 2019, Turkey [51]	Syria	Qual.	N = 10 (21-28), *n* = 5 female, *n* = 5 male; refugees	Convenience	2.5 – 5 years
Dolezal, 2021, USA [52],	South Asia, Middle East, North Africa, Europe, Central Asia	Quant.	N = 92 (18-44), 28.6 % (*n* = 32) female, 72.4% (*n* = 69); refugees /migrants	Convenience	-
Dowling, 2021, Australia [53]	Afghanistan, Pakistan, Iraq, Syria	Qual.	N = 19 (20-59), 79% (*n* = 15) female, 21% (*n* = 4) male; refugees	Convenience	Less than four years
Ersahin, 2020, Turkey [54]	Syria	Quant.	N = 805, 19-77, 45.57% (*n* = 383) females, 40.8% (*n* = 329 males); refugees	Convenience	-
Ferriss and Forrest-Bank, 2018, Kenya [55]	Somalia	Qual.	N = 12 (18+), 50% (*n* = 6) female, 50% (*n* = 6) male; refugees	Purposive, snow-ball	1.3-15 years
Flothmann, 2021, UK [56]	Africa, Middle East, Central Asia	AR	N = 9 (20-59), *n* = 1 female, *n* = 8 male; refugees	Purposive	-
Gal and Hanley, 2020, Israel [57]	Argentine	Qual.	N = 15 (48-55); migrants	Purposive	-
Goodman et al., 2017, USA [58]	Mexico, Central / South America, Middle East, Africa	Qual.	N = 19 (mean: 35.5, SD = 8.3, range 26-62), female; refugees	Purposive, snow-ball	0.4–18.0 years, M = 5.2, (SD = 5.9)
Gruttner, 2019, Germany [59]	Diverse	Quant.	N = 995 (21-26), 20% (n = 199 female), 80% (*n* = 796) male, migrants	Convenience	-
Hartonen V., 2021, Finland [60]	Arabic countries, Turkey, Iraq, Iran, Somalia	MM	N = 181 (17+), 21.5% (*n* = 39) female, 78.5% (*n* = 142) males; refugees	Convenience, snow-ball	-
Hussain and Bhushan, 2013, India [61]	Tibet	Qual.	N = 12 (mean: 35, SD = 6.5, range 25-46), 33% (*n* = 4) female; 67% (*n* = 8) male; refugees	Snow-ball	N = 5 born in exile, *n* = 7 born in Tibet
Hussain and Bhushan, 2011, India [62]	Tibet	Quant.	N = 226 (mean: 43.96, SD = 15.46), 33% (*n* = 74) female, 65% (*n* = 152) male; refugees	Purposive	-
Jibeen and Khalid, 2010, Jibeen, 2011, Canada [63], [64]	Pakistan	Quant.	N = 308 (mean: 35.8, SD = 7.31, range 25-50), 47% (*n* = 31) female, 57% (*n* = 176) male; migrants	Purposive	1-5 years (mean 3.4, SD = 1.36)
Jibeen, 2019, Pakistan [65]	Afghanistan	Quant.	N = 137 (mean = 35.81, SD = 7.75, range 25-50) male; refugees	Purposive	Mean 26.36, (SD = 10.25).
Kim and Lee, 2009, South-Korea [66]	North-Korea	Qual.	N = 5 (20-39), n = 3 female, n = 2 male; refugees	Purposive	6 months – 6 years
Kuttikat M, 2018, India [67]	Sri Lanka	Qual.	N = 15 (23-54), 40% (*n* = 6) female, 60% (*n* = 9) male; refugees	Purposive	Arrived 1984, 1990, 2006
Muruthi, 2020, Thailand [68]	Burma	Qual.	N = 14 (18-60), *n* = 6 women, *n* = 8 men; migrants	Convenience	-
Lavie-Ajayi and Slonim-Nevo, 2017, Israel [69]	Sudan	Qual.	N = 8 (27 – 38), male; refugees	Convenience	4 – 7 years
Lee, 2020, USA [70]	Ecuador, Dominican Republic, Mexico, Colombia, Peru	Quant.	N = 306 (mean 38, range: 18 – 80), 52.6% (*n* = 160 female), 47.4% (*n* = 146 male); migrants	Random	-
Lenette et al., 2013, Australia [71]	Sudan, Burundi, Congo	Qual.	N = 4 (range 30-50) female; refugees	Purposive, snow-ball	2-5 years
Lim and Han, 2016, South Korea [72]	North Korea	Quant.	N = 445 (mean 40, SD = 12.0), 76.0% (*n* = 338) female, 24% (*n* = 107); refugees	Random	Less than 4 years
Liu, 2020, Canada [73]	Syria, Iraq, Afghanistan, Iran, Kenya, Vietnam, Somalia, Mexico	Qual.	N = 21 (mean 36; SD = 53.5), *n* = 10 female, *n* = 11 male; refugees	Convenience/ snowball	8<5 years, 13 >5 years
Maria, 2021, Greece [74]	Different countries	Quant.	N = 64 (mean 35.72, SD = 7.75), *n* = 23 females, N = 40 males; refugees	Purposive	-
Mahonen et al., 2013, Finland [75]	Russia	Quant.	N = 224 (mean 44.4, SD = 15.0, range 19-85), 68.3% (*n* = 152) females; 32.7% (*n* = 72); migrants	Purposive / language classes	At baseline 3 – 15 months
Maung et al. 2021 [76], USA	Burma	Qual.	N = 11 (mean 35, SD = 12, range 22-57), female; refugees	Purposive	-
Melamed et al., 2019, Switzerland [77]	Eritrea	Qual.	N = 10 (median 28.5; range 20-35), males; refugees	Purposive	18-36 months
Mwanri, 2021, Australia [78]	Kenya, Nigeria, Zambia, Tanzania, Ghana, Zimbabwe, South Africa, Rwanda	Qual.	N = 27 , *n* = 12 female, *n* = 15 male; refugees	Convenience	-
Mera-Lemp, 2020, Chile [79]	Latin-America	Quant.	N = 194 (mean: 34,77, SD = 10.181, range 18-67), 50% (*n* = 97) female, 50% (*n* = 97) male; migrants	Convenience	-
Nam et al., 2016, South Korea [80]	North Korea	Quant.	N = 380 (mean: 40.95, SD = 8.98), 66.2% (*n* = 200) female, 33.8% (*n* = 102) male; refugees	Random	Mean stay 63.54 months
Nashwan et al., 2019, USA [81]	Iraq	Qual.	N = 22 (mean: 54.7), female; refugees	Purposive, snowballing	1-4 years
Novara et al., 2021, Italy [82]	Africa, Asia, Europe	Quant.	N = 354 (mean :33.30, SD = 11.9), 48.4% (*n* = 171) female, 51.6% (*n* = 183) male; refugees	Convenience	-
Nyarko et al., 2021 [83], Ghana	Liberia	Qual.	N = 12 (range: 25-35); refugees	Convenience	-
Obrist and Buchi, 2008, Switzerland [84]	Africa	Qual.	N = 20 (range:33-46), *n* = 9 female, *n* = 11 male; refugees	Convenience	-
Ogtem-Young, 2018, United Kingdom [85]	Azerbajan, India, Iraq, Iran, Pakistan, Turkey	Qual.	N = 18 (range: 25– 63), *n* = 4 females, *n* = 11 males; migrants	Purposive, snowball	-
Paloma et al., 2014, Spain [86]	Marroco	Quant.	N = 633 (mean: 31.9, SD = 8.5), 51.8% (*n* = 343) female, 48.2% (*n* = 290); migrants	Convenience	1-59 years
Pearce, 2017, Canada [87]	Sudan	Qual.	N=8, female; refugees	Purposive	-
Penman, 2017, Australia [88]	England, India, Pakistan, Papua New Guinea, the Philippines, Portugal, South Africa	Qual.	N = 10, *n* = 7 female, *n* = 3 male; migrants	Convenience	2-5 years
Poudel-Tandukar et al., 2019, USA [89]	Bhutan	Quant.	N = 225 (mean age: 37.6, SD = 14.5, age range 20-65), 49.8% (*n* = 112) female, 50.2% (*n* = 113) male; refugees	Purposive	3-5 years
Rizkalla and Segal, 2018, Jordan [90]	Syria	Quant.	N = 250 (mean age 35.74, SD = 11.20, range 16-75); 54.6% (*n* = 136) female, 45.4% (*n* = 114) male; refugees	Purposive	Mean 14.32 months
Roth and Ekblad, 2006, Sweden [91]	Kosovo	Quant.	N = 218, 56% (*n* = 122) female, 44% (*n* = 116) male; refugees	Convenience	3 and 6 months
Simich and Andermann, 2014, Canada [92]	Sudan	Qual.	N = 30 (age range: 20-60); refugees	Snow-ball	2000 - 2003
Simkin, 2020, Israel [93]	Latin-america	Quant.	N = 204 (age range: 18-80), 65.2% (*n* = 133) female, 34.8%, *n* = 71 male; migrants	Purposive	-
Simsir, 2021, Turkey [94]	Syria	Qual.	N=15 (18-40), *n* = 10 female, *n* = 5 male; refugees	Snow-ball	1-12
Skalisky, 2020, Jordan [95]	Syria, Palestine	MM	N = 110, n = 38 males (35%), 65% (*n* = 71), (mean: 35, SD = 12.21), 35% (*n* = 39); refugees	Purposive	-
Smit and Rugunanan, 2015, South Africa [96]	Congo, Burundi, Zimbabwe	Qual.	N = 50 (age range 22-48), female; refugees	Purposive	2-10 years
Solberg, 2021, Sweden [97]	Afghanistan, Eritrea, Iraq, Somalia,				
Syria, stateless	Quant.	N = 455 (18-64), 26.8% (*n* = 122) females, 73.2% (*n* = 333) males; refugees	Purposive	4,5% prior to 2014; 8.6% 2016-2018	
Sossou et al., 2008, USA [98]	Bosnia	Qual.	N = 7 (32-47), female; refugees	Purposive	10-12 years
Ssenyonga, 2013, Congo [99]	Uganda	Quant.	N = 426 (mean age: 35.11, SD = 12.64), 51.6%% (*n* = 220 females), 49.4% (*n* = 206) males; refugees	Random	Refugee camp
[Subedi et al., 2019, Canada 100]	Bhutan	Quant.	N = 109 (18+), 48.6% (*n* = 48) female, 49.4% (*n* = 61) males; refugees	Convenience	Since 2015
Taher, 2020, UK [101]	Syria	MM	N = 154, 42.6% (*n* = 23) females, 57,4% (*n* = 54 males); refugees	Convenience	-
Taylor, 2020, UK [102]	Nigeria, Guinea, Iran, Sierra Leone. Congo, Liberia, Zimbabwe	Qual.	N = 12 (28-61), *n* = 9 female, *n* = 3 male; refugees	Convenience	5-21 years
Thomas-Taylor and Cerulli, 2011, Australia [103]	Pakistan, Somalia	Qual.	N = 101 (median 60, age range 60-92); refugees	Convenience, purposive	-
Tippens, 2017, Kenya [104]	Congo	Qual.	N = 55 (18-70), 50.9% (*n* = 28) female, 49.1% (*n* = 27) male; refugees	Purposive	-
Tippens et al., 2021, USA [105]	Iraq	Qual.	N = 9; refugees, female and male	Purposive	2.75-21
Tonsing, 2020, USA [106]	Burma	Quant.	N = 204, mean: 35.76, (SD = 11.3), 52.0% (*n* = 106) female, 48.0% (*n* = 98 male); refugees	Purposive	-
Udah, 2019, Australia [107]	Different countries in Africa	Qual.	N = 30, *n* = 10 females, *n* = 20 males; refugees / migrants	Purposive, snowball	< 3 years
Udwan, 2020, Netherlands [108]	Syria	Qual.	N = 22, *n* = 12 female, *n* = 10 male, 18-38; refugees	Purposive, snowball	-
Uy and Okubo, 2018, USA [109]	Cambodia	Qual.	N = 12 (mean: 54.5%, range: 33-81), 4 female, *n* = 8 male; refugees	Purposive, snowball	< 20 years
Young, 2018, USA [110]	Burma	Qual.	N = 14 (N = 6 female, *n* = 8 male), 18-60; refugees	snowball /purposive	-
Walther et al., 2021, Germany [111]	Syria, Afghanistan	Qual.	N = 54 (N = 24 female, *n* = 30 male), 18-55; refugees	Convenience / Snowballing	Arrived 2013-2018
Welsh and Brodsky, 2010, USA [112]	Afghanistan	Qual.	N = 8 (mean: 43, SD = 15.5, range: 20-73), female; refugees	Snowball sampling	<1981-2001

**TABLE 3:** Outcomes, studies, promotive and preventive factors in qualitative and in quantitative studies included in the review. Global, 2021.

Outcome	Promotive and preventive factors in more than one of the studies	
	Quantitative studies	Qualitative studies
Resilience	Hope, religion, forgiveness, spirituality, income, cultural coping strategies, self-efficacy, family, employment, education, strength, ego power, flexibility, energy, self-confidence, humor, giving family support, receiving social support (friends, family, social services)	Hope, focus on the future, religion, trust, family, appropriation of stress as an illness concept, humor, cultural heritage, determination, family support; borrowing networks; active forgetting, families;, caring for other, opportunities to work and self –educate, caring for children; community, helping others, ingenuity, past war experiences; religion, language, circles of support, global community, giving/receiving social support
Sense of coherence	Perceived control (longer time in a refugee camp)	Perceived control, migration stress, religion, social connections, taking responsibility, help from NGOs
Posttraumatic growth	Hope, cognitive coping, values before flight, meaningful relationships, personal strength, religiosity, satisfaction with perceived social support, drive to overcome difficulties, positive outlook, ability to find meaning in adversity, faith, culture, traditions, supportive relationships, family, forgiveness, income, acceptance, connectedness, PTSD, providing help	Hope, strength, determination, religion, interdependent relationships, family relations, education, helping others, acceptance
Mental Wellbeing	Fulfillment of premigration expectation, social justice in the new country and individual strengths, engagement in forward –focus coping strategies, expectations, education, employment, cognitive coping; orientations towards integration, resilience, belonging; migration, religion	Self-support, religion, strong relationship with child, forming friendships, education, hope for the future, being independent, contributing to society, faith, religion, family, friends, community support, future orientation, language, friendship, community building

### Region of Study and Origin of Participants

In most of the studies (*n* = 60), the transnational migrants were defined as being refugees (Table 1). In the rest of the studies, participants were defined as migrants (*n* = 13), or as refugees (including asylum seekers) and migrants (*n* = 7).

### Study Sample Characteristics

The study sample sizes ranged from *n* = 50 [35] to *n* = 995 [59] among quantitative studies, and from *n* = 4 [71] to *n* = 55 [104] among qualitative studies (Table 2). The majority of the participants were female (57.21%, *n* = 5,748) and the age range of the participants across all studies ranged from 18–68 years. The participants originated from more than 30 countries including Afghanistan [65, 111, 112], Congo [104], Eritrea [34, 113], Iran [73, 85], Iraq [60, 81, 85, 105, 114], Somalia [39, 115, 116], and Syria [38, 51, 53, 54, 73, 90, 94, 95, 101, 108, 111, 117] (Table 1).

Most of the studies were conducted in high income countries such as Australia [43, 53, 71, 78, 88, 107, 116, 118], Canada [63, 64, 87, 100], and the USA [35–37, 39, 40, 42, 47, 50, 52, 58, 70, 76, 81, 89, 98, 106, 109, 110, 112], with fewer being conducted in low and middle income countries: in Congo [99], Chile [79], Ghana [119], India [62], Jordan [38, 90, 95], Kenya [55, 104], South - Korea [66, 80, 120], Nepal [121], South Africa [96], Thailand [68], South-Africa [96] and Turkey [45, 48, 51, 54, 94]. Three studies reported that their participants were living in refugee camps [65, 69, 119], while the participants of the remaining studies were living in the community.

### Study Sampling Methods

The quantitative studies generally used purposive sampling or convenience sampling methods, however, three studies used random sampling [70, 72, 99] (Table 2). The qualitative studies used the following sampling methods: purposive (*n* = 20), combined methods (*n* = 15). convenience (*n* = 9) or snowballing (*n* = 6). The review findings on determinants of resilience, PTG, SOC are presented separately according to study design (Supplementary Tables S1–S3).

### Quantitative Studies Exploring Promotive and Preventive Factors of Resilience, SOC and Mental Wellbeing

The 30 quantitative studies included in the review constituted *n* = 8,651 participants. The sample sizes of the quantitative studies ranged from *n* = 50 [35] to *n* = 995 [59], with a mean size of *n* = 288 participants (Table 1). Resilience was measured using self-designed questionnaires [122] or versions of the Resilience Scale (RS-25, RS-11, RS-8) [89, 123], or the Connor-Davidson Resilience Scale (CD-RISC) [45]. PTG was measured using the Post-traumatic Growth Inventory (PTGI) [35, 62, 90], and SOC using the Sense of Coherence Scale [91]. Additionally, we identified studies investigating mental wellbeing. Mental wellbeing was measured using the BBC wellbeing scale [36], and the General Wellbeing Schedule [47, 100], among others (Supplementary Table S6).

### Qualitative Studies Exploring Promotive and Preventive Factors of Resilience, SOC or Mental Wellbeing

The *n* = 39 qualitative studies identified in the review included *n* = 749 transnational migrants (Table 2). The study sample sizes ranged from *n* = 4 to *n* = 55. The following methods were applied in the studies: focus groups discussions [34, 37, 50, 55, 96, 124], individual interviews [40, 43, 50, 51, 53, 57, 58, 62, 68, 73, 76–78, 81, 83–85, 88, 94, 96, 104, 109, 116, 118, 125], participatory research [56, 87], and photovoice [42]. Additionally, some studies used ethnographic methods [42, 71], or direct observations [71, 77, 81, 84, 98].

### Risk of Bias Assessment

Most of the studies were assigned a low risk of bias and determined to be of high quality. The main sources of potential risk of bias among quantitative studies were the lack of information on non-responders (all studies), or no justification of the sample size (*n* = 25) (Supplementary Table S5). Among the qualitative studies, a moderate rating was assigned to four studies that did not explicitly address the relationship between researcher and participants or ethical aspects [41] (Supplementary Table S5). No study was assigned a high risk of bias.

### Measurement of Adversities

The measurement and definition of adversity exposure was heterogeneous across studies (Tables 2, 3). While some studies used a variety of self-report measures to assess adversity, others used standardized measures such as the Communal Traumatic Events Inventory, the Language, Identity and Behavioral Acculteration Scale (LIB), Harvard Trauma Questionnaire (HTQ), Refugee Trauma Experience Inventory (RTEI), Multidimensional Acculterative Stress Scale (MASS), Psychological Trauma Scale, Family the Adaptability and Cohesion Evaluation Scale (FACES-II), and the War Event Questionnaire (WEQ) (Supplementary Table S3).

### Promotive and Preventive Factors

Results from the included studies are summarized in Table 3 and discussed hereafter according to the key categories of influencing factors identified by the studies. A variety of factors associated with resilience, PTG, SOC and wellbeing were identified in both quantitative and qualitative studies. Since some of the factors identified in both study types overlapped, the findings are summarized together. The main influencing factor at the individual level was hopefulness and future orientation. Hopefulness was described as desire accompanied by expectation to be able to fulfill the desire in the host country was identified in quantitative and in qualitative studies [35, 41, 69, 98, 109, 112, 126]. Future orientation was described as determination, and forward-oriented coping [35, 36, 86]. Further influencing factors at the individual level were religiousness [34, 37, 39, 58, 100], and spirituality [66, 71, 104, 127]. Support received by family [37, 55, 72, 80] and being able to support family members [96, 112, 116, 128] and friends [42, 55, 69, 129] were reported to be important factors at the relationship level, while financial resources [45, 65, 71, 90] and access to work [69, 75, 100] and education [65] played a role at the societal level (Table 3).

## Discussion

This paper examined factors influencing resilience, PTG, SOC and mental wellbeing among transnational migrants. Studies identified by the search varied widely regarding population samples, context, study design, measurements, approach to data analysis, and whether the primary study focus was on resilience or mental health. Therefore, a narrative synthesis approach was adopted to capture this heterogeneity. In both quantitative and qualitative studies, individual (forward-orientation and hope, spirituality and religiousness), relationship (caring and belonging), as well as societal factors (opportunities for education and employment, opportunities for advocacy and activism) were reported to contribute towards resilience, PTG, SOC and mental wellbeing. The findings across the included studies were relatively consistent, despite the studies being set in diverse and varied contexts across different countries and with participants of diverse cultural backgrounds and migration experiences. Taken together resilience in transnational migrants was influenced by individual factors (e.g., forward orientation and hope. religiousness), family factors (e.g., caring and belonging), community factors (e.g., peer support) and society factors opportunities for education, work, advocacy and activism.

### Methdological Factors

Methodological factors in both quantitative and qualitative studies were not related to the outcome.

### Promotive and Preventive Factors

#### Forward-Orientation and Hope

The individual factors were mainly future oriented, such as hope and forward-orientation. Hope has been conceptualized as state, as trait and as process. Hope as positive motivational cognitive-emotional process is activated during times of difficulty and is a component of individual adaptability and agency. As such hope is a multidimensional process which has emotional, cognitive, motivational, social and identity related components. Hope can be understood in a more individualist way or as context dependent. People with high hope are more likely to perceive a situation as controllable and manageable; they usually find solutions more quickly compared to individuals with low hope [130]. Synder portrayed hope as a goal-oriented cognitive construct with affective and behavioral implications [130]. Hence, hope predicts progress towards goal attainment and functions as an important resource to enhance resilience. Hope, accordingly, includes planning and motivation and the expectation that positive outcomes will occur through a person`s agency. Hope has been linked to positive moods, wellbeing, adjustment, resilience and trust. One of the studies including refugees from South Sudan who had resettled in Uganda indicated that the refugees would not have left their home countries had circumstances not forced them to do so, because of the distant hope of peace and security (Meyer 2019). In another study, hope was found to be related to positive outcome perspectives in the life of migrants (Stone 2018). In line with this research, hope and accomplishment of goals reciprocally affect each other. Conversely, when people sense that they are not making progress, their tendency to engage in agency thinking might be reduced.

#### Spirituality and Religiousness

Positive religious coping includes religious forgiveness, seeking spiritual support, and reappraising God as benevolent. Religiousness might constitute engaging in religious activities, which was observed to provide a sense of normality to participants in the studies. This suggests that attempting to generate a state that feels normal, comfortable, or predictable, as perceived by the individual, may also be a strategy that some transnational migrants adopt.

#### Caring and Belonging

Social connectedness - where people experience a sense of belonging, is a well‐established protective factor for mental health. Consistent with our findings, family bonding was listed as one of the most commonly reported factors in a recent systematic review [131]. This emphasis on the importance of family relationships is in line with the idea that transnational migrants’ resilience encompasses a more communal notion of resilience than the Western, more individualized concept.

#### Opportunities for Education and Employment

A further factor identified in the quantitative and qualitative studies concerns opportunities to learn and work. Education and employment opportunities have been shown to influence the integration process of transnational migrants in their new societies [132]. Accordingly, the post-migration situation, including discrimination and inability to work and study due to restrictions, may negatively impact resilience. The findings regarding perceived opportunities are in line with prior research demonstrating the effects of post-migration, e.g., stress on migrants’ mental health due to poor employment opportunities. Income is a powerful determinant of health and affects mental health in every age group. A meta-analysis of 59 studies comparing refugee mental health to that of resident populations revealed a linear relationship between refugees’ mental health and the right to work, access to employment, and socioeconomic status. Though studies included in the meta-analysis did not assess visa type or authorized legal status, the findings indicate that economic opportunities are a critical factor for resilience.

#### Opportunities for Prosociality (Advocacy and Caring)

Furthermore, prosocial behavior and the perception of being able to contribute, care and to be active in the host country was observed to be critical for resilience in the studies included in our review. This finding indicates that the social determinants of mental health apply to international migrants’ resilience and post-traumatic growth, and the impact of social inclusion and exclusion on resilience can be measured in quantitative studies and is likewise perceived in qualitative studies. Further, the findings suggest that psychosocial services for migrants should address these factors of providing opportunities for advocacy or activisms. Giving transnational migrants opportunities for prosociality might contribute to the feeling of belonging to the host society.

#### Strengths and Limitations

The strength of this review is that, to our knowledge, it is the first to bring together and synthesize studies on factors contributing to resilience, PTG, SOC and mental wellbeing. Despite these strengths, this review is not without limitations. Studies were excluded if the migration status of the participants was unclear or not reported. Therefore, potentially relevant studies may have been excluded. Overall, the current evidence base is limited mostly to cross-sectional studies, making it difficult to draw causal relationships between the factors identified and the outcomes. Further, the qualitative findings are based on self‐reported narratives and are subject to common limitations across all the studies such as social desirability, under reporting, and relying on memory. The evidence base would benefit from longitudinal studies to better understand factors that promote positive outcomes during migration.

The different study designs and methods applied, especially the diverse sampling, restrict the cross-applicability of findings and makes generalizations difficult. Conducting research with transnational migrant populations is associated with many challenges, one of which is sampling. While restricting the inclusion criteria to studies that incorporated multi-stage representative sampling might have further improved the quality of the review, this would have greatly reduced the number of studies fitting the criteria. A further limitation might be related to the tools used to measure the outcomes of interest. Although most of the tools had been widely used in different cultural contexts, none had been specifically developed for migrants.

### Conclusion

To conclude, this review provides evidence on the positive role of hope and prosociality on transnational migrants’ resilience trajectories. One of the implications of this study is that rather than perceiving refugees as ‘passive victims’ suffering from mental health problems, attention should be given to the resilience of transnational migrants and the factors contributing to resilience. Based on the results of this study, we can conclude that there are modifiable factors which can contribute to resilience. Focusing on resilience and PTG rather than trauma is crucial in shifting the portrayal of victimized transnational migrants and instead encourages policies and psychosocial services tailored towards giving transnational migrants, especially forced migrants opportunities and higher autonomy.

The focus on a purely psychological model of impact of migration may be an obstacle to adopting a more cultural appropriate Public health approach towards migration, that includes resilience and responding to adversities with hope and prosociality. Concepts of responding to adversities that focus on dealing with the past are not necessarily applicable to the livelihood of transnational migrants in their search for making peace with the past. By shifting the focus to the strengths and capacities of individuals who migrate, this form of research can promote a view of these individuals as capable, resourceful, motivated persons, who persevere amidst adversities. Such research is also crucial as a basis for the creation of new policies, programs, and interventions that can benefit migrants in general. Research, policy, and practice are often focused on documenting vulnerabilities rather than strengths*.* Findings from this review suggest that programs and resource allocation should be directed to areas that encourage or facilitate hope and opportunities for migrants to enable a future‐orientated focus.
