# Supplementary material for: Factors Contributing to Resilience Among First Generation Migrants, Refugees and Asylum Seekers: A Systematic Review
Source: Int J Public Health. 2023 Dec 11;68:1606406. doi: 10.3389/ijph.2023.1606406 (PMC10749365; doi:10.3389/ijph.2023.1606406)
Supplement: Supplementary file 1 [file Table1.docx]

**Supplemental material**

**Table 1. Overview and summary of quantitative study findings**

| **Author, year, country** | **Sample size, country of origin** | **Outcomes** | **Outcome measure(s)** | **Adversity** | **Adversity measure** | **Confounder** | **Confounder**  **measure** | **Summary of significant associations** |
| --- | --- | --- | --- | --- | --- | --- | --- | --- |
| Ai et al., 2007, USA [34] | N=50, Kosovo | PTG, PTSD | SRGS, PSS | War related trauma | CTEI | Education, hope, coping | Own scale, multidimensional coping scale; hope scale | Coexistence of PTSD and PTG; hopefulness and cognitive coping predicts PTG |
| Aikawa & Kleyman, 2019, USA [35] | N=90, Southeast Asia, Africa | Well-being, trauma-focus-, forward focus coping | BBC Well-Being Scale , PACT | Migration process | - | Age, gender, marital status, length of stay in US, social support, coping strategies | Own scale, satisfaction with support, PCI | Forward-focus coping, community support, flexible coping, associated with well-being (F (3,77)=5.17, p=0.003), physical wellbeing (F (1,79)=11.86, p=0.001), psychological wellbeing (F (1,79)=4.84, p=0.031) relationship wellbeing F (1,79)=11.21, p=0.001) |
| Alduraidi et al., 2020, Jordan [37] | N=151, Syria | Resilience | CD-Risk | - | - | Age, gender, living place, employment | Own scale | Living in camps associated with higher resilience (t_144,_  p=0.027), better education associated with resilience |
| Areba et al., 2018, USA [38] | N=156, Somalia | Well-being,  depression, anxiety | HSCL-25, SF-12 | History of migration | Own scale | Age, gender, employment, religious coping | Own scale, 14-item Brief COPE Scale | Religious coping |
| Braun-Lewensohn et al., 2019, Greece [43] | N=111, Syria | Individual, community sense of coherence | SOC-scale | War related events | Own question | Gender, age, education, | Own scale | Longer time in the camp predicts better SOC |
| Cengiz et al., 2019, Turkey [44] | N=310, Syria | Resilience, PTG, PTSD | CD-Risk, PTG-I, HTQ | War related events, traumatic stress | Harvard Trauma Questionnaire (HTQ), IES | Gender, age, education, employment, income, marital status, cigarette use, alcohol use, residence | Own scale | PTSD, financial, social resources associated with PTG; PTG higher among refugees with PTSD |
| Cetrez et al., 2021, Sweden [45] | N=410, Iraq | Resilience, meaning of life, perceptions of illness, PTSD | CD-Risk, own scale, EMIC, PC-PTSD screen | Trauma experience, everyday worries | Own scale | Gender, age, marital status, education, employment, economy, physical safety, language proficiency, religion, social support | Own scale | Parents / siblings social support; religion; adaptation |
| Christopher, 2000), USA [46] | N=100, Ireland | Resilience, well being | RSA, GWB | Migration | - | Gender, age, education, employment, marital status, number of health care appointments | Demographic and Migration Questionnaire (DMQ) | Higher resilience (β= 0.22, p=0.02) and greater satisfaction (β= 0.52, p=0.000) predict wellbeing (β= -0.19,p=0.03) |
| Civan Kahve et al., 2020, Turkey [47] | N=101, Iraq | Resilience, PTSD | RSA | Migration events | Migration history, basic needs during migration | Age, gender, humor | Own scale | Social support, high self-perception, ego power, flexibility, energy, self-confidence, humor |
| Dolezal, 2021, USA [51] | N=102, South Asia, Middle East, North Africa, Europe, Central Asia | PTG, quality of life | PTGI, SF 12 | Perceived discrimination | EDS-R | Gender, age, education, social connectedness | Own measure | Social connectedness (sense of belonging) associated with PTG |
| Ersahin, 2020, Turkey [53] | N=805, Turkey | PTG, coping, PTSD | PTGI, PTSD | War events | HTQ. IES, Brief Cope | Demographic information (gender, age, ethnicity, education level, socio  economic status, income level), religiosity | Own measure | Higher levels of PTSD predicted higher levels of PTG, religiosity |
| Gruttner, 2019, Germany [58] | N=995, Diverse | WHO 5 Short Scale | Well-being | Belonging, worries about discrimination | Own scale | Social resources, belonging | Own measure | Belonging, integration into the host country |
| H Hussain & Bhushan, 2011, India [61] | N=226, Tibet | PTG, Post-traumatic stress | PTGI, Impact of Event Scale (IES) | Traumatic events | RTEI | Age, sex, education, marital status, generation, family type, emotion regulation | Own measure, CERQ | PTSD: B: .500 (p<.01), putting into perspective: B: 297; refocus on planning: B: .196 |
| Jibeen & Khalid, 2010, Jibeen, 2011, Canada [62, 63] | N=308, Pakistan | Psychological wellbeing, sense of coherence | Psychological well-being scale, SOC, general health questionnaire | Acculturation stress | MASS | Age, gender, education, income, comfort level, nature of job, duration of stay in Canada, number of friends), mi-gration information, coping, social support | Own measure, Brief Cope scale, MSPSS | Acculturative stress (B: .442; income (B: -.110), job relevance (B: -.099), perceived social support (B:-143), emotion focussed strategies |
| Jibeen, 2019, Pakistan [64] | N=137, Afghanistan | Well-being, sense of coherence | Well-being scale , SOC | Refugee situation | MASS | Age, gender, education, income, length of stay in Pakistan, perceived control, social support, affects, stress, coping | Own measure, MSPSS, PANAS, Acculturative Stress Scale, Brief COPE | Perceived control:  B=-0.15; acculterative stress: B=0.14 |
| Lee, 2020, USA [69] | N=306, Ecuador, Dominican Republic, Mexico, Colombia, Peru | Resilience | CD-Risk | Loneliness | Own scale | Gender, country of origin, language, age, education, social support income, immigration status, length of time in the US | Demographic information sheet, MSPSS | Loneliness  significantly associated with resilience  (B = − 0.11, 95% CI − 0.21, 0.00, *p* < .05). |
| Maria, 2021, Greece [73] | N=64, different countries | PTSD, Resilience | HTQ, CD-Risk, | Migration experiences | - | Depression, Religion, personal characteristics | PHQ-9, Own scale | Religion is associated with resilience |
| Mahonen et al., 2013, Finland [74] | N=224, Russia | Well-being,  life satis-faction | GWBI, own scale | Pre migration experiences | - | Expectations about future life | Own scale, | Expectations which were exceeded life satisfaction B: 0.35, p< .001 |
| Mera-Lemp, 2020, Chile [78] | N=194, Latin-America | Well-being | Ryffs Well-being Scale | Acculturation stress | Immigrant Acculturation Scale | Gender, age, education, household income | Own scale | Psychological well-being associated with orientations of integration |
| Nam et al., 2016, South Korea [79] | N=380, North Korea | Resilience, depression | CD-Risk, CES-D | Family functioning | FACES-II | Gender, age, education, house-hold income, number of family members residing together, time in South Korea, traumatic experiences | Own scale | Family cohesion associated with resilience (B: 0.92, p<0.001), traumatic experience: B:4.33, p=0.04) |
| Novara et al., 2021, Italy [81] | N=354, Africa, Asia, Europe | Well-being, resilience | Satisfaction with Life Scale, CD-Risk | General health | Own scale | Age, gender, marital status, sense of community | Own scale, sense of community index | Resilience has an impact on well-being, belonging |
| Paloma et al., 2014, Spain [85] | N=633, Morocco | Well-being | Satisfaction with Life Scale | Political conservativism, cultural sensitivity, residential segregation | Own scales | Use of coping strategies, satisfaction with the receiving context | Own scale | Well-being associated with cultural sensitivity (B: 0.56 (95% CI 0.21-0.91); use of active coping strategies: B: 0.11 (95% CI 0.3-0.19), satisfaction with the receiving context: B: 0.09 (95% CI 0.05-0.13) |
| Poudel-Tandukar et al., 2019, USA [88] | N=225, Bhutan | Resilience, anxiety, depression | 25-item resilience scale, HSCL–25 | Forced migration, flight related traumatic events | Own scales | Age, gender, marital status, employment, years residing in the US; Coping style, social support, alcohol consumption | Own measure, CSI-SF, MSPSS | Resilience inversely associated with anxiety (beta = -0.026; p = .037), depression (beta = -0.036, p = .041) |
| Rizkalla & Segal, 2018, Jordan [89] | N=250, Syria | PTG, PTSD, distress | PTGI, HTQ, K6 | War events | WEQ | Age, gender, years of marriage, education, number of children, months in camps | Own scale, HTQ | PTG/ wellbeing associated with income (r = .34, beta = .26, p = .001), NGO assistance (r=.07, beta=.14, p=.045); absence of affective disorders / psychosis |
| Roth & Ekblad, 2006, Sweden [90] | N=218, Kosovo | Sense of coherence general health, depression/ anxiety | SOC-12, GHQ, HSCL-25 | Trauma, post migration stress | Own measure | Postmigration stress | Duration of stay | Post migration stress associated with decreased SOC |
| Simkin, 2020, Israel [92] | N= 204, Latin-America | Well-being, satisfaction with life | SWLS | Migration | Own measure | Age, education, country of origin, length of stay, centrality of event, spirituality | Own scale, CES, ASPIRES-SF | Migration, religion and spirituality |
| Solberg, 2021, Sweden [96] | N=455, Afghanistan, Eritrea, Iraq, Somalia,  Syria | Well-being, coping | Well Being Index, WHO-5, Brief Cope | Post-migration stress, coping, family conflicts | RPMS | Age, education, country of origin, length of stay | Data from the Swedish Migration Board, on measure | Cognitive restructuring had positive effects; perceived discrimination, distressing family conflicts had adverse effects on well-being. |
| Ssenyonga, 2013, Congo [98] | N=426, Congo | Resilience | CD-Risk, PTG, PDS | War experiences | - | Age, education | Own scale | Availability of family support, age, previous displacements |
| Subedi et al., 2019, Canada [99] | N=109, Bhutan | Well-being, Coping | GWB, Brief COPE | - | - | Age, education, employment, religion, stay in host country | Own scale | College level of education: B=0.25 (95% CI: 0.55-13.45), positive reframing: B=0.21 (95% CI 0.52-6.60) |
| Tonsing, 2020, USA [105] | N=204, Burma | Resilience | BRICS | Social support | MSPSS | Age, education, employment, social support, distress | Own scale, K-10 | Support from family and friends |

**Abbreviations:** ABS: Affect Balance Scale; ASPIRES-SF: Assessment of Spirituality and Religious Sentiments Scale,; BRICS: Brief Resilient Coping Scale; CES: Brief Centrality of Event Scale; CTEI: Communal Traumatic Events Inventory; HSCL-25: Hopkins Symptoms Checklist-25, GWB: General Well-being Schedule, TBDI: Talbieh Brief Distress Inventory, FACES-III: Family functioning was measured by Korean version of Family Adaptability and Cohesion Evaluation Scale, K-CD-RISC: The 25-item Korean version of the Connor-Davidson Resilience Scale , CES-D: Korean version of the Center for Epidemiologic Studies Depression, HSCL-25: Hopkins Symptom Checklist-25, CSI-SF: 32-item Coping Strategies Inventory–Short Form; K10: Kessler Psychological Distress Scale; MASS: Multidimensional Acculturative Stress Scale-24 item, MSPSS: 12-item Multidimensional Scale of Perceived Social Support, GHQ-28: General Health Questionnaire, SCO-12: BSI: Brief Symptom Inventory, LOT: the 12- item Life Orientation Test, GWBI: The General Well-Being Index (GWBI) of Gaston and Vogl (2005); PACT: Perceived Ability of Coping with Trauma Scale; PTGI-SF= Posttraumatic Growth – Short Form; RFQ: Regulatory Focus Questionnaire; SWLS: Satisfaction with Life Scale; SRGS: Stress-Related Growth Scale, PSS: Posttraumatic Symptom Scale¸ Proactive Coping Inventor; RSA: Resilience Scale for Adults; RTEI: Refugee Trauma Experience Inventory, FACES-II: Family Adaptability and Cohesion Evaluation Scale, PMSS: Post-Migration Stress Scale.
