# Supplementary material for: Factors Contributing to Resilience Among First Generation Migrants, Refugees and Asylum Seekers: A Systematic Review
Source: Int J Public Health. 2023 Dec 11;68:1606406. doi: 10.3389/ijph.2023.1606406 (PMC10749365; doi:10.3389/ijph.2023.1606406)
Supplement: Supplementary file 2 [file Table2.docx]

**Supplemental material**

**Table 2. Overview and summary of qualitative study findings**

| **Author, year, country** | **Sample size (age), country of origin** | **Data collection methods** | **Main outcome** | **Adversity** | **Other outcomes** | **Analysis** | **Key findings** |
| --- | --- | --- | --- | --- | --- | --- | --- |
| Abraham et al., 2018, Norway [33] | N=18 (18-60), Eritrea | Focus groups, individual interviews | Resilience, coping, PTG | War; experiences before, during and after migration | Strength, resilience, PTG | Content focused hermeneutic analytic approach | Focus on the future, religion, trust, psychological problems considered as normal, optimism, fellowship with and support from fellow Eritrean refugees |
| Akinsulure-Smith, 2017, USA [36] | N=38 (19-68), Cameroon, CAR, Gabon, Guinea, Liberia, Mali, Senegal, Sierra Leone | Focus groups | Coping strategy, resilience | Migration, undocumented migrant status | Distress, intimate partner violence, parenting, extended-family demands | Grounded theory methodology | Humor, cultural heritage, local peer support, religion, family |
| Atari-Khan, 2021, USA [39] | N=8 (27-59), Syria | Individual interviews | Resilience | Experiences of forced migration | Prefight-, flight-, post flight experiences | Thematic analysis | Family and community tangible support; caring for family, faith, hope for a better future, establishing roots |
| Babatunde-Sowole et al., 2020, Australia [42] | N=22 (18+), West-Africa | Individual interviews | Resilience | Experiences of forced migration | Prefight, flight, post flight experiences | Thematic analysis | Ability to source income, ingenuity, creating safe environments, faith/reliosity, sense of belonging |
| Baird, 2012; Baird & Boyle, 2012, USA [40,41] | N=10 (25-44), Sudan | Interpretive ethnography | Well-being | Experiences of forced migration | Linearity, self-support, hope for future | Interpretive ethnography | Self-support, hope for the future; religion/spirituality |
| Copping et al., 2010, Australia [48] | N=15 (19-49), Sudan | Individual interviews | PTG | Protracted conflict situation in Sudan, living in a refugee camps | Trauma, distress | Grounded theory methodology | Support that they both give and receive, religion, new possibilities, hope, own strength, determination, new possibilities |
| Corley & Sabri, 2021, USA [49] | N=39, Eritrea, Sudan, Uganda, Ethiopia, Kenya, Congo, Ghana | Individual interviews, focus groups | Resilience | Political violence, armed conflict, intimate partner violence, post-migration events | Political violence, armed conflict | Thematic analysis | Religion, artistic expression, exercise, mental health care, family / friend support |
| Demir, 2019, Turkey [50] | N=10 (21-28), Syria | Individual interviews | Resilience | - | - | Phenomenological  analysis | Social contribution, career goals, patience, desire for learning, self-confidence, desire for learning, grit, spirituality, financial situation, host society support, community/family support |
| Dowling, 2021, Australia [52] | N=19 (20-59), Afghanistan, Pakistan, Iraq, Syria | Individual interviews | Well-being | Migration | Worries about loved ones | Interpretive phenomenological analysis | Faith, religion, family/ friends/community social support, future orientation, freedom, safety |
| Ferriss & Forrest-Bank, 2018, Kenya [54] | N=12 (18+), Somalia | Focus group discussions | PTG | Post-migration acculturation stress | Acculturative stress, social isolation | Thematic analysis | Cultural values, perseverance, religion, interdependent relationships, family relation |
| Flothmann, 2021, UK [55] | N=9 (20-59), Africa, Middle East, Central Asia | Participatory research | Well being | Migration | Resilience | Thematic analysis | Religion, social connections, language, friendship, community building |
| Gal & Hanley, 2020, Israel [56] | N=15 (48-55), Israel | Individual interviews | Resilience | - | Mental well-being | Form and content analysis | Political activism |
| Goodman et al., 2017, USA [57] | N=19 (26-62), Mexico, Central /South America, Middle East, Africa | Individual interviews | Resilience | Migration, Family separation, employment, economic status | PTSD, depression, suicidality | Interpretive phenomenological analysis | Reliance on government supports, faith and positive beliefs about the opportunities in the  United States (e.g., safety and education for their children) |
| Hussain & Bhushan, 2013, India [60] | N=12 (25-46), Tibet | Individual interviews | Resilience | Traumatic experiences | Coping | Interpretive phenomenological analysis, content analysis | Religion, community bonding and support, historical examples of stress and resiliency |
| Kim & Lee, 2009, South-Korea [65] | N=5 (20-39), Korea | Individual interviews | PTG | Persecution, starvation, war,  imprisonment | Recovery factors | Interpretive phenomenological analysis | Family, friends, emotional professional support, religion |
| Kuttikat M, 2018, India [66] | N=15 (23-54), Sri Lanka | Group interviews | Resilience | Loss, confusion, discrimination | - | Grounded theory | Religion, social connections, organizational help education, taking responsibility, practical solutions |
| Lavie-Ajayi & Slonim-Nevo, 2017, Israel [68] | N=8 (27-38), Sudan | Group interviews | Resilience | War, traumatic experience | Cognitive coping strategies | Analytic induction  and constant comparison analyses | Hope, cognitive coping, ability to work and study, support of family and friends, social and political activism |
| Lenette et al., 2013, Australia [70] | N=4 (30-50); Sudan, Burundi, Congo | Participant observation, in-depth interviews, visual ethnography | Resilience | Human rights violations | Daily life stress | Intersectional approach | Overcoming poverty and disadvantage, education, language; inspiration and confidence from past experiences; prayer, religion |
| Liu, 2020, Canada [72] | N=21; Syria, Iraq, Afghanistan, Iran, Kenya, Vietnam, Somalia, Mexico | Individual interviews | Resilience | Immigration | Coping | Thematic analysis | Female gender, education, experience with past challenges, religion, positivity, acceptance, self-care, identity, proactivity, adaptability; support, employment |
| Maung et al. 2021, USA [75] | N=11 (22-57); Burma | Individual interviews | Resilience | - | PTG | Consensual qualitative research (CQR) | Social support, emotional support with community members, hopefulness, future orientation, personal self-care, religion, cognitive coping |
| Melamed et al., 2019, Switzerland [76] | N=10 (20-35); Eritrea | Individual interviews and RS-14 | Resilience | - | Mental health | Thematic framework analysis | Ability to plan for the future, access capital, religious faith, vision for the future, family support, voluntary work, social support, contact to NGOs, religious faith, being involved in voluntary work |
| Mwanri, 2021, Australia [77] | N=27; Kenya, Nigeria, Zambia, Tanzania, Ghana, Zimbabwe, South Africa, Rwanda | Individual interviews | Resilience | Migration | Social capital | Thematic framework analysis | Religion, faith, hope, community oriented attitudes, interpersonal connections, capacities to work, family support networks, cultural traditions |
| Muruthi, 2020, Thailand [67] | N=14 (18-60); Thailand | Individual interviews | Resilience | - | Overcoming adversity | Thematic analysis | Religion, family |
| Nyarko et al., 2021, Ghana [82] | N=12 (25-35)  Liberia | Individual interviews | Resilience | War related experiences | Future orientation | Content analysis | Past adversities, future orientation, determination to transform one`s life, optimisms |
| Nashwan et al., 2019, USA [80] | N= 22; Iraq | Individual interviews | Resilience | - | Mental health | Existential-phenomenological approach | Security, safety, family; meeting expectations |
| Obrist & Buchi, 2008, Switzerland [83] | N=20 (33-46); Subsahara | Individual interviews | Resilience | Migration related adversities | Personal Growth | Thematic analyses | Health, active coping strategies, engagement in social groups, visits to the country of origin, training/education, active help seeking from professionals, stress as concept |
| Ogtem-Young, 2018, United Kingdom [84] | N=18 (25-63); Azerbaijan, India, Iraq, Iran, Pakistan, Turkey | Individual interviews | Resilience | War, traumatic experiences, racism | Adjusting to a new country | Thematic analyses | Faith |
| Pearce, 2017, Canada [86] | N=8 , Sudan | Participatory action research (PAR) | Resilience | War, traumatic experiences |  | Thematic analyses | Faith/spirituality, future orientation, beauty, giving and receiving support, taking care of one another, belonging |
| Penman, 2017, Australia [87] | N=10, Australia | Individual interviews | Resilience | - | Coping | Thematic analyses | Self-efficacy, goal focusing, adapting, hope |
| Simich & Andermann, 2014, Canada [91] | N=30 (2-60), Sudan | Individual interviews | Resilience, mental well-being | War, traumatic experiences, resettlement | - | Inductive approach | Emotional support from family, fulfilling social roles, meeting social expectations, solving problems, dignity and growth |
| Simsir, 2021, Turkey [93] | N=15 (18-40); Syria | Individual interviews | PTG | War, traumatic experiences | - | Content analysis | Studying, religion/spirituality, patience, hope for the future, adaptation, social support |
| Smit & Rugunanan, 2015, South Africa [95] | N=50 (22-48); Congo, Burundi, Zimbabwe | Focus groups | Resilience | Conflicts in Congo, Burundi | - | Constant comparative method; phenomenological analysis | Caring for others, contributing to family income, asking for help, spirituality, determination, hope, love / concern for children |
| Sossou et al., 2008, USA [97] | N=7 (32-47); Bosnia | Individual interviews | Coping strategies | War in Bosnia | Resilience | Narrative analysis | Self-determination, optimism, inner strength, hope, family, spirituality |
| Taylor, 2020, UK [101] | N=12 (28-61); Nigeria, Guinea, Iran, Sierra Leone. Congo, Liberia, Zimbabwe | Individual interviews | Resilience | War, loss | PTG | Interpretative phenomenological analysis | Gratitude, faith/religiosity, helping others, acceptance |
| Thomas-Taylor & Cerulli, 2011, Australia [102] | N=24 (60-92); Pakistan, Somalia | Individual interviews, Photo voice focus group discussions | Well-being | Persecution, torture, migration experiences | Coping | Thematic analysis | Relationships with supportive friends/family members; being able to volunteer, coping ability |
| Tippens, 2017, Kenya [103] | N=55 (18-70), Congo | In-depth interviews; participant observation | Well-being, resilience | Exposure to war, displacement | Resilience | Iterative thematic analyses | Faith in God’s plan, trust in religious community, establishing borrowing networks, compart-mentalizing the past and present. |
| Tippens et al., 2021, USA [104] | N=9, Iraq | Photo voice | Resilience | War related events | Trauma, cultural bereavement | Thematic analysis | Religion, cultural rituals, language |
| Udah, 2019, Australia [106] | N=30, Different countries in Africa | In-depth interviews | Resilience | Migration related events | Vulnerability | Thematic analysis | Employment, expanding capabilities, future orientation, family, faith, opportunities |
| Udwan, 2020, Netherlands [107] | N=22 (18-38), Syria | In-depth interviews | Resilience | - | - | Thematic analysis | Social support from peers, family, social services, work, study, cooking, entertainment, contemplation, emotion management |
| Uy & Okubo, 2018, USA [108] | N=12 (33-81), Cambodia | In-depth interviews | PTG | Genocide related events | Posttraumatic growth | Interpretive phenomenological analysis (IPA) | Sharing trauma narrative, work, hope, optimism, education, community activism, pursuit to fulfil a “survivor’s mission” |
| Walther et al., 2021, Germany [110] | N=54 (18-55), Afghanistan, Syria | Individual interviews | Resilience |  | Mental health | Thematic analysis | Social support present / future orientation, opportunities for learning and personal development, belonging, having children, young age, education, volunteering |
| Welsh & Brodsky, 2010, USA [111] | N=8 (20-73), Afghanistan | Individual interviews | Resilience | War experiences | Mental health | Thematic analysis, member check, consultation with key informants | Problem-focused/active coping, helping others, family support, hope, focus on future, gratitude, determination, religion |
| Young, 2018, USA [109] | N=14 (18-60), Burma | Individual interviews | Resilience | Migration | Health | Thematic analysis | Giving/receiving support, culture |
