# Supplementary material for: Factors Contributing to Resilience Among First Generation Migrants, Refugees and Asylum Seekers: A Systematic Review
Source: Int J Public Health. 2023 Dec 11;68:1606406. doi: 10.3389/ijph.2023.1606406 (PMC10749365; doi:10.3389/ijph.2023.1606406)
Supplement: Supplementary file 3 [file Table5.docx]

**Supplemental material.**

**Table 5. Risk of bias of qualitative studies included in the review on factors contributing to resilience**

|  | 1 | 2 | 3 | 4 | 5 | 6 | 7 | 8 | 9 | 10 |
| --- | --- | --- | --- | --- | --- | --- | --- | --- | --- | --- |
|  | Was there a clear statement of the aims of the research? | Is a qualitative methodology appropriate? | Was the research design appropriate to address the aims of the research? | Was the recruitment strategy appropriate to the aims of the research? | Was the data collected in a way that addressed the research issue? | Has the relationship between researcher and participants been adequately considered? | Have ethical issues been taken into consideration? | Was the data analysis sufficiently rigorous? | Is there a clear statement of findings? | How valuable is the research? |
| Abraham et al., 2018, Norway [33] | Y | Y | Y | Y | Y | N | Y | Y | Y | Y |
| Akinsulure-Smith, 2017, USA [36] | Y | Y | Y | Y | Y | N | Y | Y | Y | Y |
| Atari-Khan, 2021, USA [39] | Y | Y | Y | Y | Y | N | CT | Y | Y | Y |
| Babatunde-Sowole et al., 2020, Australia [42] | Y | Y | Y | Y | Y | N | Y | Y | Y | Y |
| Baird, 2012; Baird & Boyle, 2012, USA [40,41] | Y | Y | CT | Y | Y | N | CT | Y | Y | CT |
| Copping et al., 2010, Australia [48] | Y | Y | Y | Y | Y | N | CT | Y | Y | Y |
| Corley & Sabri, 2021, USA [49] | Y | Y | CT | Y | Y | N | CT | Y | Y | Y |
| Dowling, 2021, Australia [52] | Y | Y | Y | Y | Y | N | Y | Y | Y | Y |
| Demir, 2019, Turkey [50] | Y | Y | Y | Y | Y | N | CT | Y | Y | Y |
| Ferriss & Forrest-Bank, 2018, Kenya [54] | Y | Y | Y | Y | Y | Y | Y | Y | Y | Y |
| Flothmann, 2021, UK [55] | Y | Y | Y | Y | Y | Y | Y | Y | Y | Y |
| Gal & Hanley, 2020, Israel [56] | Y | Y | CT | Y | Y | N | Y | Y | Y | Y |
| Goodman et al., 2017, USA [57] | Y | Y | Y | Y | Y | Y | CT | Y | Y | Y |
| Hussain & Bhushan, 2013, India [60] | Y | Y | Y | Y | Y | N | Y | Y | Y | N |
| Kim & Lee, 2009, South-Korea [65] | Y | Y | Y | Y | Y | N | N | Y | Y | Y |
| Kuttikat M, 2018, India [66] | Y | Y | Y | Y | Y | Y | Y | Y | Y | Y |
| Lavie-Ajayi & Slonim-Nevo, 2017, Israel [68] | Y | Y | N | Y | CT | N | Y | Y | Y | Y |
| Lenette et al., 2013, Australia [70] | Y | Y | Y | Y | Y | N | CT | Y | Y | Y |
| Liu, 2020, Canada [72] | Y | Y | Y | Y | Y | Y | Y | Y | Y | Y |
| Maung et al. 2021, USA [75] | Y | Y | Y | CT | Y | N | CT | Y | Y | Y |
| Melamed et al., 2019, Switzerland [76] | Y | Y | N | Y | Y | N | Y | Y | Y | N |
| Muruthi, 2020, Thailand [67] | Y | Y | Y | Y | Y | Y | Y | Y | Y | Y |
| Mwanri, 2021, Australia [77] | Y | Y | Y | Y | Y | Y | Y | Y | Y | Y |
| Nashwan et al., 2019, USA [80] | Y | Y | Y | Y | Y | Y | Y | Y | Y | Y |
| Nyarko et al., 2021, Ghana [82] | Y | Y | Y | Y | Y | N | Y | Y | Y | Y |
| Obrist & Buchi, 2008, Switzerland [83] | Y | Y | Y | Y | Y | N | Y | Y | Y | N |
| Ogtem-Young, 2018, United Kingdom [84] | Y | Y | Y | Y | Y | N | Y | Y | Y | Y |
| Pearce, 2017, Canada [86] | Y | Y | Y | Y | Y | N | Y | Y | Y | Y |
| Penman, 2017, Australia [87] | Y | Y | Y | Y | Y | Y | Y | Y | Y | Y |
| Simich & Andermann, 2014, Canada [91] | Y | Y | Y | Y | Y | N | Y | Y | Y | Y |
| Simsir, 2021, Turkey [93] | Y | Y | Y | Y | Y | N | N | Y | Y | Y |
| Smit & Rugunanan, 2015, South Africa [95] | Y | Y | N | Y | Y | N | Y | Y | Y | Y |
| Sossou et al., 2008, USA [97] | Y | Y | Y | Y | Y | N | Y | Y | Y | Y |
| Taylor, 2020, UK [101] | Y | Y | Y | Y | Y | Y | Y | Y | Y | Y |
| Thomas-Taylor & Cerulli, 2011, Australia [102] | Y | Y | Y | Y | Y | N | Y | Y | Y | Y |
| Tippens, 2017, Kenya [103] | Y | Y | Y | Y | Y | Y | Y | Y | Y | Y |
| Tippens et al., 2021, USA [104] | Y | Y | Y | Y | Y | Y | Y | Y | Y | Y |
| Udah, 2019, Australia [106] | Y | Y | Y | Y | Y | N | Y | Y | Y | Y |
| Udwan, 2020, Netherlands [107] | Y | Y | Y | Y | Y | N | Y | Y | Y | Y |
| Uy & Okubo, 2018, USA [108] | Y | Y | Y | Y | Y | Y | CT | Y | Y | Y |
| Walther et al., 2021, Germany [110] | Y | Y | Y | Y | Y | Y | Y | Y | Y | Y |
| Welsh & Brodsky, 2010, USA [111] | Y | Y | Y | Y | Y | Y | CT | Y | Y | Y |
| Young, 2018, USA [109] | Y | Y | Y | Y | Y | N | Y | Y | Y | Y |
