# Supplementary material for: Factors Contributing to Resilience Among First Generation Migrants, Refugees and Asylum Seekers: A Systematic Review
Source: Int J Public Health. 2023 Dec 11;68:1606406. doi: 10.3389/ijph.2023.1606406 (PMC10749365; doi:10.3389/ijph.2023.1606406)
Supplement: Supplementary file 4 [file Table3.docx]

**Supplemental material.**

**Table 3. Overview and summary of mixed methods studies**

| **Author, year, country** | **Country/ region of origin** | **Country of immigration** | **Study design** | **Participants** | **Outcome** | **Findings** |
| --- | --- | --- | --- | --- | --- | --- |
| Taher, 2020, UK [100] | Syria | UK | Mixed methods | Refugees | PTG | Valuing  others more, pursuing a new career path, discovering inner strength,  appreciation of life and detachment from it, strengthened belief. |
| Hartonen V., 2021, Finland [59] | Arabic, Kurdish, Somalia | Finland | Mixed methods | Refugees | Well-being | Hope and safety |
| Skalisky, 2020, Jordan [94] | Syria, Palestine | Jordan | Mixed methods | Refugees | Resilience (CD-Risk) | Access to education, positive religious coping |
