# Supplementary material for: Factors Contributing to Resilience Among First Generation Migrants, Refugees and Asylum Seekers: A Systematic Review
Source: Int J Public Health. 2023 Dec 11;68:1606406. doi: 10.3389/ijph.2023.1606406 (PMC10749365; doi:10.3389/ijph.2023.1606406)
Supplement: Supplementary file 5 [file Table4.docx]

**Supplemental material.**

**Table 4. Risk of bias of quantitative studies included in the review on factors contributing to resilience**

|  |  | 1 |  | 2 | 3 | 4 | 5 | 6 | 7 | 8 | 9 | 10 | 11 |  | 12 | 13 | 14 | 15 | 16 |  | 17 | 18 | 19 | 20 |
| --- | --- | --- | --- | --- | --- | --- | --- | --- | --- | --- | --- | --- | --- | --- | --- | --- | --- | --- | --- | --- | --- | --- | --- | --- |
|  | **Introduction** | Were the aims/objectives of the study clear? | **Methods** | Was the study design appropriate for the stated aim(s)? | Was the sample size justified? | Was the target/reference population clearly defined? (Is it clear who the research was about?) | Was the sample frame taken from an appropriate population base so that it closely represented the target/reference population under investigation? | Was the selection process likely to select subjects/participants that were representative of the target/reference population under investigation? | Were measures undertaken to address and categorise non-responders? | Were the risk factor and outcome variables measured appropriate to the aims of the study? | Were the risk factor and outcome variables measured correctly using instruments/measurements that had been trialled, piloted or published previously? | Is it clear what was used to determined statistical significance and/or precision estimates? (e.g. p-values, confidence intervals) | Were the methods (including statistical methods) sufficiently described to enable them to be repeated? | **Results** | Were the basic data adequately described? | Does the response rate raise concerns about non-response bias? | If appropriate, was information about non-responders described? | Were the results internally consistent? | Were the results presented for all the analyses described in the methods? | **Discussion** | Were the authors' discussions and conclusions justified by the results? | Were the authors' discussions and conclusions justified by the results? | Were there any funding sources or conflicts of interest that may affect the authors’ interpretation of the results? | Was ethical approval or consent of participants attained? |
| Ai et al., 2007, USA [34] |  | Y |  | Y | Y | Y | Y | DK | N | Y | Y | Y | Y |  | Y | DK | DK | Y | Y |  | Y | Y | DK | DK |
| Aikawa & Kleyman, 2019, USA [35] |  | C |  | Y | N | Y | Y | DK | N | Y | Y | Y | Y |  | Y | DK | DK | Y | Y |  | Y | Y | DK | DK |
| Alduraidi et al., 2020, Jordan [37] |  | Y |  | Y | N | Y | Y | N | N | Y | Y | Y | N |  | Y | DK | DK | N | Y |  | Y | Y | N | Y |
| Areba et al., 2018, USA [38] |  | Y |  | Y | N | Y | Y | DK | N | Y | Y | Y | Y |  | Y | DK | DK | Y | Y |  | Y | Y | N | Y |
| Braun-Lewensohn et al., 2019, Greece [43] |  | Y |  | Y | Y | Y | Y | N | N | Y | Y | Y | Y |  | Y | DK | DK | Y | Y |  | Y | Y | N | Y |
| Cengiz et al., 2019, Turkey [44] |  | Y |  | Y | N | Y | Y | DK | N | Y | Y | Y | Y |  | Y | DK | DK | Y | Y |  | Y | Y | DK | DK |
| Cetrez et al., 2021, Sweden [45] |  | Y |  | Y | N | Y | Y | N | N | Y | Y | Y | Y |  | Y | DK | DK | Y | DK |  | Y | Y | N | Y |
| Christopher, 2000), USA [46] |  | Y |  | Y | N | Y | Y | DK | N | Y | Y | Y | Y |  | Y | DK | DK | Y | Y |  | Y | Y | N | DK |
| Civan Kahve et al., 2020, Turkey [47] |  | Y |  | Y | N | Y | Y | N | N | Y | Y | Y | Y |  | Y | DK | DK | Y | Y |  | Y | Y | DK | Y |
| Dolezal, 2021, USA [51] |  | Y |  | Y | N | Y | Y | N | N | Y | Y | Y | Y |  | Y | DK | DK | Y | Y |  | Y | N | N | Y |
| Ersahin, 2020, Turkey [53] |  | Y |  | Y | N | Y | Y | DK | N | Y | Y | Y | Y |  | Y | DK | DK | Y | DK |  | Y | N | DK | Y |
| Gruttner, 2019, Germany [58] |  | Y |  | Y | N | Y | Y | N | N | Y | Y | Y | Y |  | Y | DK | DK | Y | Y |  | Y | Y | N | Y |
| H Hussain & Bhushan, 2011, India [61] |  | Y |  | Y | N | Y | Y | DK | N | Y | Y | Y | N |  | Y | DK | DK | N | DK |  | Y | Y | DK | DK |
| Jibeen & Khalid, 2010, Jibeen, 2011, Canada [62, 63] |  | Y |  | Y | N | Y | Y | DK | N | Y | Y | Y | Y |  | Y | DK | DK | Y | Y |  | Y | Y | DK | Y |
| Jibeen, 2019, Pakistan [64] |  | Y |  | Y | N | Y | Y | DK | N | Y | Y | Y | N |  | Y | N | N | N | DK |  | Y | Y | N | Y |
| Lee, 2020, USA [69] |  | Y |  | Y | N | Y | Y | Y | N | Y | Y | Y | Y |  | Y | DK | DK | Y | Y |  | Y | Y | N | Y |
| Lim, 2016 |  | Y |  | Y | Y | Y | Y | DK | N | Y | Y | Y | Y |  | Y | DK | DK | Y | Y |  | Y | N | N | Y |
| Mahonen et al., 2013, Finland [74] |  | Y |  | Y | N | Y | Y | Y | N | Y | Y | Y | Y |  | Y | DK | DK | N | Y |  | Y | Y | DK | DK |
| Mera-Lemp, 2020, Chile [78] |  | Y |  | Y | Y | Y | Y | N | N | Y | Y | Y | Y |  | Y | DK | DK | Y | Y |  | Y | Y | DK | Y |
| Maria, 2021, Greece [73] |  | Y |  | Y | N | Y | Y | N | N | Y | Y | Y | Y |  | Y | DK | N | Y | Y |  | Y | Y | N | Y |
| Nam et al., 2016, South Korea [79] |  | Y |  | Y | N | Y | Y | DK | N | Y | Y | Y | Y |  | Y | DK | DK | Y | Y |  | Y | Y | N | DK |
| Novara et al., 2021, Italy [81] |  | Y |  | Y | N | Y | Y | N | N | Y | Y | Y | Y |  | Y | DK | DK | Y | Y |  | Y | Y | DK | DK |
| Paloma et al., 2014, Spain [85] |  | Y |  | Y | N | Y | Y | Y | N | Y | Y | Y | Y |  | Y | DK | DK | Y | Y |  | Y | Y | N | DK |
| Poudel-Tandukar et al., 2019, USA [88] |  | Y |  | Y | N | Y | Y | DK | N | Y | Y | Y | Y |  | Y | DK | DK | DK | Y |  | Y | Y | N | Y |
| Rizkalla & Segal, 2018, Jordan [89] |  | Y |  | Y | Y | Y | Y | DK | N | Y | Y | Y | Y |  | Y | DK | DK | Y | Y |  | Y | Y | N | Y |
| Roth & Ekblad, 2006, Sweden [90] |  | Y |  | Y | N | Y | DK | DK | N | Y | Y | Y | N |  | Y | DK | DK | DK | DK |  | Y | Y | DK | Y |
| Simkin, 2020, Israel [92] |  | Y |  | Y | Y | Y | Y | N | N | Y | Y | Y | Y |  | Y | DK | DK | Y | Y |  | Y | N | N | Y |
| Solberg, 2021, Sweden [96] |  | Y |  | Y | N | Y | Y | N | N | Y | Y | Y | Y |  | Y | DK | DK | Y | Y |  | Y | Y | N | Y |
| Ssenyonga, 2013, Congo [98] |  | Y |  | Y | N | Y | Y | Y | N | Y | Y | Y | N |  | Y | DK | DK | Y | DK |  | Y | N | N | Y |
| Subedi et al., 2019, Canada [99] |  | Y |  | Y | Y | Y | Y | DK | N | Y | Y | Y | Y |  | Y | N | DK | Y | Y |  | Y | Y | N | Y |
| Tonsing, 2020, USA [105] |  | Y |  | Y | Y | Y | Y | N | N | Y | Y | Y | Y |  | Y | DK | DK | Y | Y |  | Y | Y | DK | Y |

Rating: Yes/No/Don’t Know-Comment
