# Supplementary material for: Factors Contributing to Resilience Among First Generation Migrants, Refugees and Asylum Seekers: A Systematic Review
Source: Int J Public Health. 2023 Dec 11;68:1606406. doi: 10.3389/ijph.2023.1606406 (PMC10749365; doi:10.3389/ijph.2023.1606406)
Supplement: Supplementary file 6 [file Table6.docx]

**Supplemental material.**

**Table 6. Measures used in quantitative studies to measure post-traumatic growth, resilience, mental well-being, sense of coherence**

| **Outcome** | **Measure** | **Short - Name** | **Domains** | **Number items** | **Authors** |
| --- | --- | --- | --- | --- | --- |
| Post-traumatic growth | 50-items Stress Related Growth Scale | SRGS | Positive changes in relationships, personal resources, life philosophy | 50 | Park CL, et al. 1996 (1) |
|  | Posttraumatic growth inventory | PTGI | Appreciation of life, new possibilities, personal strengths, relating to others, spiritual change | 21 | Tedeschi TG & Calhoun LG, 1996 [17] * (2) |
|  | Posttraumatic growth inventory-Short Form | PTGI-SF | Growth experiences after trauma | 10 | Cann A, et al. 2010 (3) |
| Resilience | Connor-Davidson Resilience Scale | CD-Risk | Ability to cope with changes, endure stress, develop strategies in face of stress | 25 | Kobasa SC, et al. 1982 (4) |
|  | Multidimensional Trauma Recovery and Resilience Interview and rating scale | MTRR-I, MTRR-99 | Authority over memory, Integration of memory and affect, Affect tolerance and regulation, Symptom mastery and positive coping, Self-esteem, Self-cohesion, Safe attachment, and Meaning making | 135/99 | Harvey M, et al. 2003 (5) |
|  | 14 item Resilience Scale | RS-14 | Two parts: 1) personal competence' (17 items): self-reliance, independence, determination, resourcefulness, mastery and perseverance, 2) 'acceptance of self and life' (8 items): adaptability, flexibility and a balanced perspective of life | 14 | Wagnild G. 2009 (6) |
|  | Wagnild and Young Resilience Scale | RS | Two parts: 1) personal competence' (17 items): self-reliance, independence, determination, resourcefulness, mastery and perseverance, 2) 'acceptance of self and life' (8 items): adaptability, flexibility and a balanced perspective of life | 25 | Wagnild G. 2009 (6) |
|  | Resilience Scale for adults | RSA | Six sub dimensions: self-perception, planned future, structured style, social competence, family cohesion, social resources | 33 | Morote R, et al. 2018 (7) |
| Mental Well-being | BBC Well-Being Scale | Well-Being BBC | Psychological well-being, physical health and well-being, relationships | 23 | Kinderman P, et al. 2011 (8) |
|  | General Well Being Index | GWBI | Life satisfaction, general mood | 6 | Gaston JE, et al, 2005 (9) |
|  | Satisfaction with Life Scale | SLS | Self-perception of own life | 5 | Diener E, et al. 1985 (10) |
|  | Psychological well-being | PWB | Self-acceptance, positive relations with others, autonomy, environmental mastery, purpose in life, personal growth | 54 | Ryff CD 1989 (11) |
|  | General well-being | GWB | Anxiety, depression, general health, positive well-being, self-control, vitality | 18 | Dupuy HJ, 1997, (12)  Cramer SR, et al. 1991 (13) |
|  | Physical and emotional well-being | SF-12 V2 | Physical, mental health | 12 | Ware J JR, et al. 1996 (14) |
|  | Well being | WHO-5 Well Being Index | Mental well being | 5 | WHO 1998 (15) |

1. Park CL, Cohen LH, Murch RL. Assessment and prediction of stress-related growth. Journal of Personality. 1996;64(1):71-105. doi: 10.1111/j.1467-6494.1996.tb00815.x
2. Tedeschi RG, Calhoun LG. The posttraumatic growth inventory: measuring the positive legacy of trauma. Journal of Traumatic Stress (1996) 9:3. doi: 10.1007/BF02103658
3. Cann A, Calhoun LG, Tedeschi RG, Taku K, Vishnevsky T, Triplett KN, et al. A short form of the Posttraumatic Growth Inventory. Anxiety Stress Coping. 2010;23(2):127-137. doi: 10.1080/10615800903094273
4. Kobasa SC, Maddi SR, Stephen Kahn. "Hardiness and health: a prospective study.." Journal of Personality and Social Psychology, 42 (1982).:168-177. doi: 10.1037/0022-3514.42.1.168
5. Harvey, Mary Rose et al. “A Multidimensional Approach to the Assessment of Trauma Impact, Recovery and Resiliency.” Journal of Aggression, Maltreatment & Trauma 6 (2003): 109 - 87.
6. Wagnild, Gail. “A review of the Resilience Scale.” Journal of nursing measurement vol. 17,2 (2009): 105-13. doi: 10.1891/1061-3749.17.2.105
7. Morote R, Hjemdal O, Martinez Uribe P, Corveleyn J. Psychometric properties of the Resilience Scale for Adults (RSA) and its relationship with life-stress, anxiety and depression in a Hispanic Latin-American community sample [published correction appears in PLoS One. 2018 Apr 16;13(4):e0196139]. *PLoS One*. 2017;12(11):e0187954. Published 2017 Nov 10. doi: 10.1371/journal.pone.0187954
8. Kinderman P, Schwannauer M, Pontin E, Tai S. The development and validation of a general measure of well-being: the BBC well-being scale. Quality of Life Research. 2011;20(7):1035-1042. doi: 10.1007/s11136-010-9841-z
9. Gaston JE, Vogl L. Psychometric properties of the general well-being index. Quality of life research. 2005;14(1):71-75. doi: 10.1007/s11136-004-0793-z
10. Diener E, Emmons RA, Larsen RJ, Griffin S. The Satisfaction With Life Scale. Journal of personality assessment. 1985;49(1):71-75. doi: 10.1207/s15327752jpa4901_13
11. Ryff CD. Happiness is everything, or is it? Explorations on the meaning of psychological well-being. Journal of Personality and Social Psychology. 1989;57(6), 1069-1081.
12. Dupuy, H. J. (1978). General Well-Being Schedule (GWB). APA PsycTests. https://doi.org/10.1037/t04083-000
13. Cramer SR, Nieman DC, Lee JW. The effects of moderate exercise training on psychological well-being and mood state in women. Journal of psychosomatic research. 1991;35(4-5):437-449. doi: 10.1016/0022-3999(91)90039-q
14. Ware JRr, Kosinski M, Keller SD. A 12-Item Short-Form Health Survey: construction of scales and preliminary tests of reliability and validity. Medical care. 1996;34(3):220-233. doi: 10.1097/00005650-199603000-00003
